# Exploratory analysis of high-dose corticosteroid therapy on epileptic encephalopathy with spike-and-wave activation in sleep

**DOI:** 10.3389/fped.2024.1388008

**Published:** 2024-08-09

**Authors:** Hu Xiaoyue, Tang Hongwei, Wang Jianbiao, Ma Jingbo, Hua Ying

**Affiliations:** Department of Neurology, Wuxi Children’s Hospital, Affiliated Children’s Hospital of Jiangnan University, Wuxi, China

**Keywords:** EE-SWAS, ESES, corticosteroid therapy, influencing factor, children, predictive model

## Abstract

**Objective:**

This study aims to evaluate the therapeutic efficacy of high-dose corticosteroid therapy in children diagnosed with epileptic encephalopathy with spike-and-wave activation in sleep (EE-SWAS), investigate associated clinical indicators influencing treatment outcomes, and establish a predictive model for recurrence.

**Methods:**

Children diagnosed with EE-SWAS who received high-dose corticosteroid therapy were categorized into responder group and non-responder group. Data on clinical parameters, electroencephalogram (EEG) features, and serum cytokine levels were collected. Six months post-treatment, the effectively treated children were further stratified into recurrence and non-recurrence groups. Risk factors for poor outcomes following corticosteroid therapy were identified using univariate analysis. Multivariate logistic regression analysis was then employed to determine independent factors influencing the recurrence of corticosteroid therapy, which facilitated the development of a predictive model.

**Results:**

The study included 48 children, with 33 cases in the responder group (effective rate = 68.8%) and 15 cases in the non-responder group. The responder group exhibited an older onset age of electrical status epilepticus in sleep (ESES) and higher proportions of combined benzodiazepines (BZDs) use (*P* < 0.05). Among those responding to corticosteroid therapy, 11 cases experienced a recurrence (recurrence rate = 33.3%), while 22 cases did not. Significant differences were observed between the two groups concerning age of seizure onset, age of ESES onset, seizure frequency, atypical presentations, and concomitant frontal lobe discharges (all *P* < 0.05). Concomitant frontal lobe discharges and an earlier age of seizure onset were identified as risk factors for ESES recurrence following corticosteroid therapy. The predictive model was formulated as Logit(P) = 2.35 × presence of frontal lobe discharges—0.802 × age of seizure onset + 2.457. The Area Under the Curve (AUC) of Receiver Operating Characteristics (ROC) was 0.93, with sensitivity and specificity at 100% and 77.3%, respectively.

**Conclusion:**

High-dose corticosteroid therapy for EE-SWAS exhibited a high effective rate as well as a notable recurrence rate. Onset age of ESES and combined benzodiazepines usage correlated with therapeutic efficacy. Seizure onset age and the presence of frontal lobe discharges may hold predictive value for recurrence following corticosteroid therapy.

## Introduction

Electrical Status Epilepticus in sleep (ESES), also known as Encephalopathy related to Status Epilepticus during slow Sleep, refers to an age- dependent epilepsy syndrome characterized by cognitive regression and epileptic abnormalities during sleep (1). It has been newly designated as epileptic encephalopathy with spike-and-wave activation in sleep (EE-SWAS) or developmental EE with SWAS (DEE-SWAS) by the International League Against Epilepsy (ILAE). For clarity, we use the term ESES specifically to refer to the electroencephalogram (EEG) phenomenon associated with EE-SWAS. The spike-wave index (SWI) serves as a criterion for evaluating ESES. There is currently no unified consensus on its diagnostic criteria. Recent studies on the management of ESES suggest a critical SWI threshold of 50% (2). While ESES typically resolves before adulthood and is often self-limited, the long-term persistent epileptic discharge during a child's brain development can disrupt the slow-wave activity in normal sleep, which may interfere with the formation of neural circuits and potentially lead to cognitive impairment (3). The cognitive and language deficits caused by ESES can persist, especially in patients who have experienced ESES for extended periods (more than 3 years) (4, 5). A study involving 24 children with idiopathic ESES found that only 25% of the children fully recovered from neuropsychological disturbances after ESES remission (6). Therefore, the principles of ESES treatment aim to control seizures and effectively suppress abnormal discharges to mitigate neurodevelopmental damage in patients (7).

In 2000, Tsuru et al. (8) demonstrated substantial efficacy in treating two children with language impairment associated with Landau-Kleffner syndrome (LKS) using high-dose intravenous methylprednisolone. Follow-up investigations found that high-dose corticosteroid therapy for (D)EE-SWAS not only significantly improves cognitive functions such as language impairment but also ameliorates abnormal EEG discharges and helps control refractory seizures (9–11). In 2015, Munckhof et al. (12) evaluated the effects of various treatments on EEG and cognitive function improvements in patients, finding that corticosteroid therapy and surgery (for patients with surgical indications) were the most effective methods for treating EE-SWAS. Similarly, the Utrecht Medical Center (13) reported their experience in treating patients with EE-SWAS, finding that corticosteroid therapy was superior to other treatments [benzodiazepines (BZDs), other antiseizure medications, surgery, intravenous immunoglobulins] in achieving cognitive improvement and EEG remission.

However, high-dose corticosteroid therapy also presents several drawbacks. Firstly, it is associated with adverse reactions from corticosteroid use. Although most children can tolerate these side effects, which typically subside after discontinuing treatment, some may experience significant issues such as Cushingoid features, hirsutism, recurrent infections, and sleep disorders. These side effects can significantly affect their daily lives and learning experiences. Secondly, the relapse rate for corticosteroid therapy is relatively high, suggesting that the use of corticosteroid only temporarily improves the prognosis for children, thereby limiting the value of treatment. In clinical practice, it is also observed that some children either do not respond to high-dose corticosteroid therapy or are at a high risk of relapse. For these patients, it is crucial to either avoid the use of corticosteroid or optimize the corticosteroid treatment regimen to maximize benefits. Therefore, research into the factors that influence the efficacy of high-dose corticosteroid therapy holds significant importance.

This study focused on patients with EE-SWAS who underwent high-dose corticosteroid therapy. We analyzed treatment efficacy, recurrence rates, and related influencing factors, and constructed a predictive model for treatment relapse. The goal was to identify the most suitable patient population for this treatment approach.

## Material and methods

### Subjects

This study collected data from patients with EE-SWAS who received treatment at the outpatient and inpatient departments of Wuxi Children's Hospital from January 2016 to December 2022.

Inclusion criteria: (1) Age: 3–17 years old; (2) Confirmation of ESES on long-term EEG monitoring, with a SWI ≥50% during non-rapid eye movement (NREM) sleep; (3) All patients had varying degrees of regression in cognitive and/or behavioral functions; (4) Completion of the treatment course involving high-dose methylprednisolone therapy and sequential oral prednisone therapy, with a follow-up duration of at least six months after the whole corticosteroid therapy.

Exclusion criteria: (1) Poor compliance of patients and their parents with treatment and examination; (2) Loss to follow-up.

A total of 48 patients were included in the study, and a retrospective analysis of their clinical data was conducted. The study was reviewed and approved by the Ethics Committee of Wuxi Children's Hospital (Ethics Approval No: WXCH2015-12-004). Written informed consent was obtained from the guardians of the children.

## Study methods

### Video-EEG monitoring

A Nihon-Kohden video-EEG (Tokyo, Japan) was used to monitor the EEG of the patients for at least 4 h. The SWI was measured as the percentage of 1-s bins with ≥1 spike during NREM sleep. EEG evaluations were conducted before treatment, at the end of treatment, and every 6 months thereafter.

### Measurement of Th1/Th2/Th17 subset cytokines

Th1 cells, Th2 cells, and Th17 cells are subtypes of helper T lymphocytes. Th1 cells secrete interleukin (IL)-2, tumor necrosis factor (TNF)-α, interferon (IFN)-γ, etc. Th2 cells secrete IL-4, IL-6, IL-10, and Th17 cells mainly secrete IL-17A. These cytokines are involved in various inflammatory processes in the body. Blood samples (4 ml) were obtained from the patients before corticosteroid therapy, and serum levels of IL-2, TNF-α, IFN-γ, IL-4, IL-6, IL-10, and IL-17A were measured using flow cytometry to assist in assessing the immune status.

### Treatment protocol and efficacy criteria

Detailed clinical data of each patient were recorded, including the age of seizure onset and the age of ESES onset, gender, seizure characteristics, seizure frequency prior to corticosteroid therapy, atypical presentations (including atypical absence, negative myoclonus, or/and oropharyngeal symptoms), cognitive function, treatment, brain magnetic resonance imaging (MRI), genetic testing (24 patients underwent whole-exome sequencing). The characteristics of pre-steroid EEG and SWI were also recorded. All patients underwent routine blood, urine, and stool tests, blood biochemistry, electrocardiogram, blood pressure monitoring, chest x-ray, T cell spot test (T-SPOT.TB), and other examinations to exclude relevant contraindications. During intravenous methylprednisolone therapy, the blood glucose, blood pressure, heart rhythm, and electrolyte levels of the patients were monitored. Informed consent for intravenous methylprednisolone therapy was obtained from the guardians of each patient. Referring to Tsuru's scheme (8), patients were treated with an intravenous infusion of high-dose methylprednisolone (15–20 mg/kg daily) for 3 consecutive days. This infusion was repeated three times with a 4-day interval between treatments, after intravenous therapy, prednisolone was given orally (2 mg/kg daily for 1 month, then gradually withdrawn). The total treatment duration was 4–6 months. The efficacy of ESES treatment was classified into two levels: (1) Effective: the ESES mode disappeared or the SWI improved by ≥50% post-treatment; (2) Ineffective: SWI reduction <50% or increase. The recurrence of ESES is characterized by the reappearance of an SWI of ≥50% during an EEG reevaluation conducted at least 6 months after the initial remission of ESES following corticosteroid therapy.

### Statistical analysis

Data processing and statistical analysis were performed using SPSS 26.0 software (IBM Corp., Armonk, NY, USA). For normally distributed continuous data, $\bar{x}\pm s$ was used to represent the data, and independent *t*-tests were used for between-group comparisons. Categorical data were presented as numbers (%) and analyzed using chi-square tests. Variables with statistically significant differences were included in logistic regression analysis to identify factors influencing the recurrence of ESES following corticosteroid therapy and to construct a predictive model. The discrimination of the predictive model was evaluated by the area under the curve (AUC) of receiver operating characteristics (ROC), and the goodness of fit of the predictive model was assessed using the Hosmer-Lemeshow test. A significance level of *P* < 0.05 was considered statistically significant.

## Results

### Clinical data

Based on the clinical data, the study included 48 children diagnosed with epilepsy, comprising 29 males and 19 females. The age of seizure onset ranged from 1 to 9 years and 6 months, while ESES onset ranged from 3 to 11 years and 2 months. Each child was followed up for at least 6 months post-treatment, with an average follow-up duration of 3 years and 4 months. The study revealed that all 48 children experienced seizures. 4 suffered from status epilepticus and 15 had seizures during wakefulness. In 44 of the 48 cases, epilepsy manifested prior to the onset of ESES. The seizure semiology primarily included generalized tonic-clonic seizures (13 patients), focal seizures (26 patients), and mixed types (5 patients). At the time of ESES onset, 18 children exhibited atypical presentations, including negative myoclonus, atypical absence, and oropharyngeal manifestations such as dysarthria or excessive salivation.

**Figure 1:**
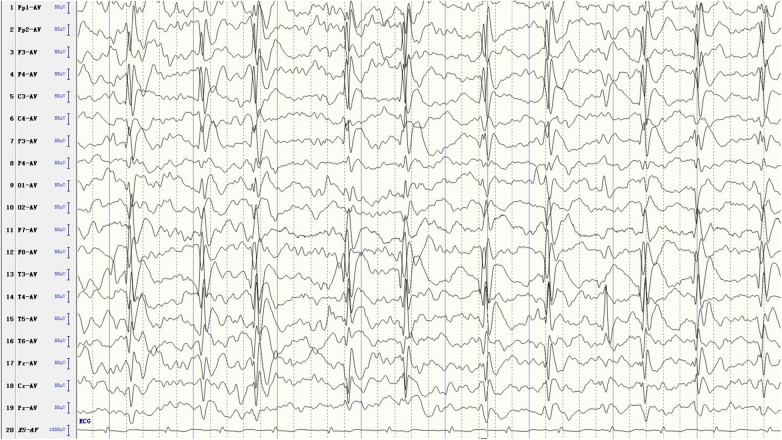
Scalp EEG of the ESES with concomitant frontal lobe discharges.

At the time of ESES diagnosis, bilateral epileptiform discharges were observed in 33 patients (68.8%), while unilateral discharges were noted in 15 patients (31.2%). The SWI was ≥85% in 14 patients (29.1%), ranged between 70% and 84% in 21 patients (43.8%), and varied from 50% to 69% in the remaining 13 patients (27.1%). The area with the most frequent and highest amplitude spikes was identified as the EEG focus. Some EEG foci were confined to the classical centrotemporal region, while others also extended to the frontal region, thus being classified as frontocentrotemporal regions (Figure 1).

Prior to receiving high-dose corticosteroid therapy, all children received antiseizure medications, including drugs such as levetiracetam (LEV), valproic acid (VPA), clonazepam (CZP), nitrazepam (NZP), and topiramate (TPM). Among them, 22 children were treated with corticosteroid therapy combined with one kind BZDs. There were no obvious abnormalities observed in blood routine tests, blood biochemistry, metabolic screening, and head MRI results in any of the children. Genetic tests were performed in 24 patients, none of them harbored known pathogenic epilepsy genes, including *GRIN2A*.

### Efficacy of corticosteroid therapy and its influencing factors

After 4–6 months of high-dose corticosteroid therapy, ESES improvement was observed as follows: 33 cases were effective, 15 cases were ineffective accompanied by refractory epilepsy, yielding an overall effectiveness rate of 68.8%. The 33 effective cases also showed improvement in their cognitive and behavioral problems, as well as reduced epileptic seizures, and were thus classified as the responder group. The remaining 15 cases were classified as non-responder group.

Comparison of clinical data, EEG features, and serum cytokine levels before corticosteroid therapy between the two groups revealed statistically significant differences in the age of ESES onset and the concomitant use of BZDs (all *P* < 0.05), as detailed in Table 1. That is, children with a later onset of ESES and concurrent use of BZDs were more likely to respond positively to high-dose corticosteroid therapy.

**Table 1:** Clinical and EEG characteristics of the responder and non-responder treatment groups.

Variable	Responder (*n* = 33)	Non-responder (*n* = 15)
Male, *n*(%）	17 (51.5%)	12 (80%)
Age at seizure onset (Mean ± SD, year)	6.00 ± 2.06	5.03 ± 1.99
Age at ESES onset (Mean ± SD, year)*	7.81 ± 2.12	6.27 ± 1.80
Seizure frequency		
≥10 times	7 (21.2%)	7 (46.7%)
Combined use of BZDs*	19 (57.6%)	3 (20%)
Status epilepticus	2 (6.1%)	2 (13.3%)
Seizures during waking phase	8 (24.2%)	7 (46.7%)
Epilepsy type prior to ESES		
Foal	18 (60%)	8 (57.1%)
Generallized	9 (30%)	4 (28.6%)
Mixed	3 (10%)	2 (14.3%)
Atypical presentations	12 (36.4%)	6 (40%)
Atypical absence		
Negative myoclonus		
Dysarthria or hypersalivation		** **
EEG findings		
SWI(%）	73.2 ± 11.9	77.0 ± 9.2
EEG lateralization		
Bilateral	25 (75.8%)	8 (53.3%)
Unilateral	8 (24.2%)	7 (46.7%)
EEG focus		
Frontocentrotemporal region	16 (48.5%)	8 (53.3%)
Centrotemporal region	17 (51.5%)	7 (46.7%)
Blood inflammatory cytokines level(pg/ml)		
IL-2	0.86 ± 0.50	0.88 ± 0.56
IL-6	1.26 ± 0.54	1.06 ± 0.78
IL-10	2.17 ± 0.77	2.42 ± 0.66
IL-17A	4.60 ± 1.99	5.00 ± 2.51
IFN-γ	1.39 ± 0.98	1.25 ± 1.09
TNF-α	0.84 ± 0.51	1.09 ± 0.68

ESES, electrical status epilepticus in sleep; BZDs, benzodiazepines; EEG, electroencephalogram; SWI, spike-wave index.

* *P* < 0.05.

In the responder group, 11 cases experienced a recurrence, with a relapse rate of 33.3%. Based on ESES recurrence, patients were divided into a non-recurrence group (22 cases) and a recurrence group (11 cases). Comparative analysis between these two groups showed statistically significant differences in the age of seizure onset, age of ESES onset, seizure frequency, atypical presentations, and concomitant frontal lobe discharges (all *P* < 0.05), as detailed in Table 2.

**Table 2:** Clinical and EEG characteristics of the relapse and non-relapse groups.

Variable	Non-relapse (*n* = 22)	Relapse (*n* = 11)
Male, *n*(%）	12 (54.5%)	5 (45.5%)
Age at seizure onset (Mean ± SD, year)**	6.86 ± 1.86	4.32 ± 1.28
Age at ESES onset (Mean ± SD, year)**	8.62 ± 1.95	6.18 ± 1.40
Seizure frequency		
≥10*	2 (9.1%)	5 (45.5%)
Combined use of BZDs	15 (63.6%)	4 (36.4%)
Status epilepticus	1 (4.5%)	1 (9.1%)
Seizures during waking phase	3 (13.6%)	5 (45.5%)
Atypical presentations**	4 (18.2%)	8 (72.7%)
Atypical absence		
Negative myoclonus		
Dysarthria or hypersalivation		
EEG findings		
SWI (%)	73.4 ± 13.0	72.7 ± 10.1
EEG lateralization		
Bilateral	16 (72.7%)	9 (81.8%)
Unilateral	6 (27.3%)	2 (18.2%)
EEG focus		
Frontocentrotemporal region*	7 (31.8%)	9 (81.8%)
Centrotemporal region	15 (68.2%)	2 (18.2%)
Blood Inflammatory cytokines level(pg/ml)		
IL-2	0.76 ± 0.35	1.08 ± 0.67
IL-6	1.19 ± 0.54	1.42 ± 0.52
IL-10	2.24 ± 0.84	2.03 ± 0.63
IL-17A	4.95 ± 2.18	3.90 ± 1.38
IFN-γ	1.54 ± 1.10	1.08 ± 0.59
TNF-α	0.91 ± 0.57	0.70 ± 0.38

ESES, electrical status epilepticus in sleep; BZDs, benzodiazepines; EEG, electroencephalogram; SWI, spike-wave index.

* *P* < 0.05.

** *P* < 0.01.

### Multiple logistic regression analysis results and construction of a predictive model for ESES recurrence after corticosteroid therapy

**Figure 2:**
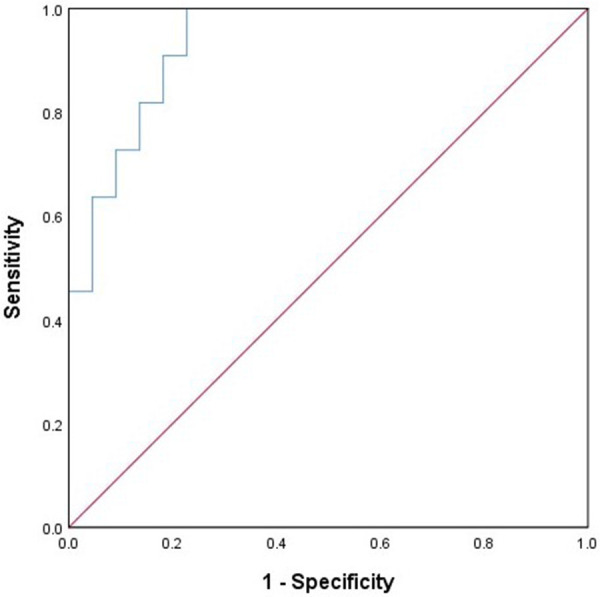
The results of the ROC curve analysis for ESES recurrence prediction model.

Through multiple logistic regression analysis, concomitant frontal lobe discharges and the age of seizure onset were identified as risk factors for ESES recurrence, as indicated in Table 3. A predictive model equation was constructed based on regression coefficients and constant terms: Logit(P) = 2.35× frontal discharge −0.802× epileptic onset age +2.457. This model was assessed via the ROC curve, showing excellent fit and predictive capability. The AUC of the model is 0.934 (95% CI: 0.855–1.000), with a sensitivity of 100% and a specificity of 77%, seen in Figure 2.

**Table 3:** Multivariate logistic regression analysis of ESES recurrence.

Variable	*β*	*P*	OR (95%CI)
Frontocentrotemporal region	2.350	0.039	10.485 (1.125–97.751)
Age at seizure onset	−0.802	0.009	0.449 (0.247–0.816)
Constant	2.457	0.187	–

### Follow-up of recurrence and follow-up treatment

**Figure 3:**
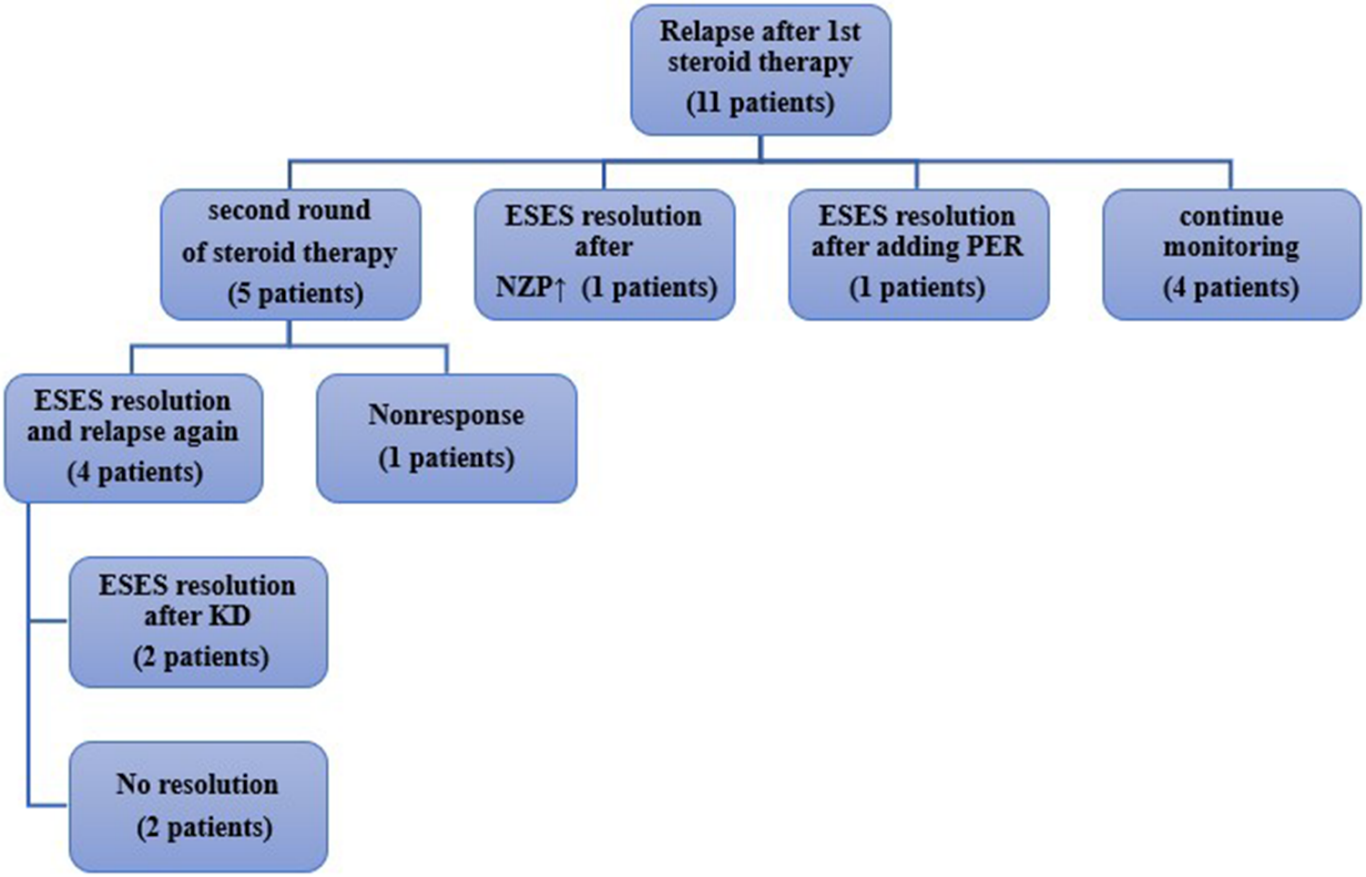
Management of ESES recurrence patients after corticosteroid therapy.

During follow-up, as shown in Figure 3, among the 11 patients with recurrence,7 experienced an increase in seizure frequency, while 4 showed only ESES recurrence on EEG. 5 patients underwent high-dose corticosteroid therapy again, of which 4 showed improvement in ESES post-treatment, while 1 showed no improvement. Among the 4 patients who responded well again to the corticosteroid therapy, 2 experienced a relapse after discontinuing the medication but improved after adopting a ketogenic diet (KD); the other 2 were given adjusted anti-seizure medications but with little effect. One patient's ESES improved after NZP dose adjustment, and another patient's ESES improved after adding perampanel(PER). Additionally, 4 children showed ESES recurrence on EEG but did not experience clinical seizures, nor was there a progressive decline in their cognitive function. Their parents opted to continue monitoring their condition.

## Discussion

### The efficacy and recurrence of high-dose corticosteroid therapy for ESES

Numerous studies have demonstrated that steroid therapy can effectively eliminate ESES, with several potential mechanisms involved (14–16): (1) Negative feedback on the hypothalamic-pituitary-adrenal axis, which lowers neuronal excitability by inhibiting the release of corticotropin-releasing hormone (CRH); (2) Neuroregulatory effects potentially include the correction of dysfunctional enzymes, alterations in intra- and extracellular electrolyte ratios, amelioration of intracellular hypoglycemia, and regulation of adenosine production within cells; (3) Enhancement of brain maturation through increased levels of brain-derived neurotrophic factor (BDNF) in cerebrospinal fluid; (4) Antiseizure effects mediated by the GABAergic system pathway. Van den Munckhof et al. (12) highlighted in their meta-analysis that the overall effectiveness of corticosteroid therapy for ESES was 81%, with a cognitive function improvement rate of 78% and an EEG improvement rate of 70%, significantly surpassing conventional antiseizure medications. In the present study, the effective rate of high-dose corticosteroid therapy for improving SWI on EEG was 68.8%. Under similar therapeutic regimens and efficacy evaluation criteria, Chen et al. (11) observed an EEG improvement rate of 82.9% following corticosteroid therapy, while Jauhari et al. (17) reported a rate of 63%, aligning with the findings of this study. However, the literature also notes that recurrence rates 1-year post-treatment can range from 33% to 89% (7, 10, 11), with the study's recurrence rates falling within that spectrum.

### Factors influencing the efficacy of high-dose corticosteroid therapy for ESES

This study delved into the relationship between various clinical characteristics, medication status, EEG indicators, and the efficacy of high-dose corticosteroid therapy in patients with ESES. The study discovered that patients with a later age of ESES onset and simultaneous use of BZDs exhibited improved effectiveness in response to high-dose corticosteroid therapy. This is consistent with the observations of Arhan et al. (18), who reported that earlier onset of ESES and longer duration of ESES were linked to a less favorable treatment outcome. A possible explanation is that the earlier the onset of epileptic seizures, the sooner ESES may manifest. This could lead to the formation of abnormal synaptic networks due to excessive neuronal excitability during the critical period of synaptogenesis, thereby resulting in a poorer response to corticosteroid therapy. Our study indicates that patients with ESES who are concurrently using BZDs exhibit an enhanced response to steroid therapy. This finding aligns with research conducted by Zhang et al. (4), which demonstrated that combining steroids with BZDs results in greater EEG improvement compared to the use of BZDs alone.

Van den Munckhof et al. (19) investigated the serum cytokine levels in 11 children with ESES and 20 healthy controls. They found that levels of IL-1α, IL-6, IL-8, IL-10, and CCL2 were significantly higher in the ESES group compared to the control group, whereas levels of MIF and CCL3 were significantly lower. Follow-up analysis of 5 children with ESES showed a significant decrease in serum IL-6 levels after immunotherapy. Changes in IL-6 were also accompanied by significant improvements in the children's EEG and neuropsychological evaluations, leading the authors to propose that IL-6 may be related to the effectiveness of immunotherapy. In our study, we also measured the levels of serum cytokines including IL-2, IL-4, IL-6, IL-10, IL-17A, TNF-α, and IFN-γ, in patients with ESES and found that the levels were within the normal range. There were also no significant differences among the treatment groups with different efficacy. The discrepancy between the two studies may mainly lie in the differences in the selection of ESES patients. In the study by Van den Munckhof et al., 5 children with ESES had abnormal head MRI results, with structural changes such as posterior cerebral artery infarction, medial temporal lobe sclerosis, extensive congenital midline defects, and focal cortical dysplasia. Perhaps the elevation of inflammatory factors is related to the underlying disease itself, as previous literature has suggested that inflammatory factors such as IL-1β and HMGB1 are overexpressed in conditions like focal cortical dysplasia, medial temporal sclerosis (20). However, all the patients in our study exhibited normal head MRI results, indicating the absence of structural abnormalities.

### Factors influencing recurrence after high-dose corticosteroid therapy for ESES and predictive model construction for recurrence

In our study, the efficacy of repeated corticosteroid therapy for patients who experienced a recurrence after initial high-dose corticosteroid therapy was unsatisfactory. Given the side effects of corticosteroid therapy, it is critically important in clinical practice to predict which patients are likely to experience a recurrence. For those with a high likelihood of recurrence after steroid treatment, exploring alternative treatments initially may be beneficial in reducing patient suffering.

Our study revealed that several factors, including a younger age at seizure onset, early onset of ESES, frequent seizures prior to corticosteroid therapy, atypical presentations, and concurrent frontal lobe discharges, are associated with a higher likelihood of recurrence after corticosteroid therapy. Zhang et al. (4) also suggested that a younger age at the onset of seizures and ESES is associated with recurrence after treatment. This may be attributed to the earlier onset of seizures and prolonged episodes, which could lead to the development of abnormal neural circuits, thus complicating treatment efforts.

Multivariate logistic regression analysis in our study suggested that a younger age at seizure onset and concomitant frontal lobe discharges are independent risk factors for recurrence. ROC curve analysis indicated that these two factors could potentially serve as predictors for the recurrence of ESES following steroid treatment. However, due to the small sample size, this predictive model requires further refinement. Previous literature has indicated that frontal lobe discharges in patients with benign epilepsy with centrotemporal spikes (BECTS) are likely to progress to ESES and are also associated with cognitive impairment (21, 22). Our findings reinforce this perspective, showing that patients with concurrent frontal lobe discharges are more likely to experience recurrence after corticosteroid therapy. This suggests a potential link between frontal lobe discharges and both the malignant progression and poor treatment outcomes of the condition.

Wiwattanadittakul et al. (7) noted that for patients experiencing recurrence, extending the tapering period of steroids may help reduce the recurrence rate. However, the potential side effects of steroids should also be taken into account. Pera et al. (23) employed a treatment regimen that involved administering high-dose methylprednisolone therapy at 15–30 mg/kg once a month over three consecutive days, with the total duration of continuous treatment extending for 4 months. In the study, 72.7% (8/11) of patients showed significant EEG improvement, similar to previous literature reports, and no significant adverse reactions to steroids or recurrences were observed during subsequent follow-ups. Although these results may be influenced by the small sample size of only 11 cases, this study suggests that this particular corticosteroid therapy may help reduce both the adverse effects and recurrence rates of steroids.

Our study also found a higher incidence of combined BZDs use among patients without recurrence, although this difference was not statistically significant when compared to the recurrence group. Additionally, increasing the dose of NZP in one of the recurrence patients also achieved ESES improvement.

## Conclusions

In summary, our study indicates that high-dose corticosteroid therapy is highly effective in treating EE-SWAS, particularly in patients with an older age at onset of ESES and those using BZDs concurrently. However, there is a notable recurrence rate associated with this treatment. The predictive model for ESES recurrence, based on the patient's age at seizure onset and concomitant frontal lobe discharges, demonstrates good predictive efficiency. This model offers a simple and reliable tool for forecasting potential relapses following corticosteroid therapy, significantly contributing to the personalized treatment of children. As this was a sing-center retrospective study, these findings will need further validation through larger samples and prospective studies.

## Data Availability

The raw data supporting the conclusions of this article will be made available by the authors, without undue reservation.
